# Videoradiographic analysis of the range of motion in unilateral experimental knee joint arthritis in rats

**DOI:** 10.1186/ar3342

**Published:** 2011-05-27

**Authors:** Michael K Boettger, Johannes Leuchtweis, Hans-Georg Schaible, Manuela Schmidt

**Affiliations:** 1 Institute of Physiology I/Neurophysiology, University Hospital, Teichgraben 8, D-07743 Jena, Germany; 2 Current address: Bayer Schering Pharma AG, Forschungszentrum Elberfeld, Aprather Weg 18 a, D-42113 Wuppertal, Germany; 3 Institute of Systematic Zoology and Evolutionary Biology, Friedrich-Schiller-University, Erbertstrasse 1, D-07743 Jena, Germany

**Keywords:** range of motion, rat, arthritis, mechanical thresholds, videoradiography, readout parameter, preclinical testing

## Abstract

**Introduction:**

The translational and predictive value of animal models highly depends on the validity of respective readout parameters. In arthritis research, there has been a shift from sole threshold testing for pain-related behavior, as well as from swelling and histology assessment for inflammation, toward an analysis of joint function as indicated, for instance, by an increasing number of studies on gait abnormalities. Clinically, the range of motion (ROM) of the affected joint plays a major role in diagnosis and the assessment of treatment benefits. This parameter, however, is only insufficiently detected by currently used analytic systems in animals.

**Methods:**

Here we used high-resolution videoradiographic analysis to assess ROM in experimental knee joint arthritis in rats. This parameter is described during the 21-day course of antigen-induced arthritis in rats. Furthermore, the therapeutic effects of antinociceptive (morphine) and anti-inflammatory (dexamethasone) treatment on ROM are documented. To obtain additional information on the implications of ROM in animal models, correlations were performed to measure pain-related behavior and inflammation.

**Results:**

The study animals showed a significant reduction in ROM of the inflamed knee joint in the acute phase of arthritis. This was accompanied by an increase in knee joint movement on the contralateral side, indicating a compensational mechanism. Both morphine and dexamethasone treatment increased and thus normalized ROM. Changes in ROM were further stage-dependently correlated with weight bearing and joint swelling, that is, with both pain-related behavior and signs of inflammation.

**Conclusions:**

The dynamic ROM observed in freely moving rats in our model of knee joint arthritis might serve as a parameter for global disease activity and might thus represent a promising readout parameter for preclinical assessment regarding the overall efficacy not only of antiarthritic but also of antinociceptive compounds.

## Introduction

Inflammatory processes in the joint are common, and a large proportion of patients with chronic pain are in fact arthritis patients [1]. While potent compounds exist to alleviate arthritis-related symptoms, a large proportion of patients remain insufficiently treated despite the use of several different therapeutic regimens. Therefore, there is a clear medical need for the development of novel antirheumatic and antinociceptive compounds.

One major problem in preclinical arthritis research is a reliable assessment of symptom severity and its alleviation by standard of care medication in respective animal models to achieve a good translational potency for predicting clinical outcome. In recent years, it has become more and more obvious that in addition to, for instance, pain threshold detection or assessment of joint swelling [2], functional parameters have great potential to identify arthritis-related surrogate parameters in animals. In this respect, readout systems have been developed that monitor locomotion in animals with defined inflammation induced in one or more joints. Parameters assessed include the speed of walking, spatial and temporal measures of step cycles (distances between pawprints, stance time, swing time and so on), the pressure and area of the pawprints and rotation in the respective limb [3-8].

One parameter which is of great clinical importance in arthritis patients is the range of motion (ROM) in the respective joint. This measure, describing the maximum flexion and extension movements in a joint from a neutral angle used for primary assessment, for describing the disease course and for estimating the efficacy of treatment. ROM measurements can be obtained at rest (static) or during movement by using videography (dynamic) [9-11]. In humans with knee joint arthritis, the respective ROM has been shown to be reduced [12,13], and this reduction furthermore correlates with the overall physical ability of patients [9]. To our knowledge, such a parameter in a respective animal model has not yet been described. Therefore, we aimed to quantify the working range of an inflamed knee joint in the model of antigen-induced arthritis (AIA) in rats and to compare this measurement to that of healthy animals. Similarly to kinematic examination in humans, we did not artificially move the joint until the animals vocalized or withdrew, but instead quantified the maximal and minimal angles between the femur and tibia in a freely moving animal (dynamic ROM). For that purpose, animals were left walking through a tunnel, and high-speed X-ray cameras filmed the movement of the skeleton at high resolution (500 frames/second). By using this method, we captured the respective angles using frame-by-frame analysis.

AIA was chosen as an immune-mediated joint inflammation whose histopathology shows many similarities to human rheumatoid arthritis [14,15]. For experimentation, the AIA model has several advantages. In immunized rats, only the AIA joint develops inflammation, thereby enabling the examination of contralateral compensational effects. Furthermore, AIA has an incidence of 100%, its acute phase starts within the first hours after antigen injection into the joint and it spontaneously progresses into chronic mononuclear inflammation with a homogeneous time course [14-17].

In addition to gaining information on the time course of this measure during the course of AIA, we aimed to distinguish between the influences of pain and mechanical factors such as swelling and joint destruction on this parameter. Hence, we included AIA animals treated with either morphine for alleviating pain or with dexamethasone as a standard anti-inflammatory agent. Furthermore, measures of primary and secondary hyperalgesia as assessed using mechanical and thermal threshold testing and measures of inflammation-induced weight shifting were obtained and correlated to the ROM.

## Materials and methods

### Induction of antigen-induced arthritis

Forty female Lewis rats (ages 6 to 8 weeks old and weighing 160 to 180 grams; Charles River, Sulzfeld, Germany) were used for the studies. AIA was induced as reported previously [15,17]. For immunization, 500 μg of antigen (methylated bovine serum albumin (mBSA); Sigma, Deisenhofen, Germany) in saline emulsified with 500 μL of Freund's complete adjuvant (Sigma) supplemented with 2 mg/mL *Mycobacterium tuberculosis *strain H37RA (Difco, Detroit, MI, USA) were injected subcutaneously twice with a one-week interval between immunizations. After another two weeks, a sterile mBSA solution (500 μg in 50 μL) was injected into the left knee joint cavity to induce monoarticular AIA in 30 rats. The results obtained from these animals were compared with those obtained from the remaining 10 rats, in which the latter intraarticular injection was omitted. These animals are referred to as "controls" below. All experiments were approved by the Thuringian state authorities (registration number 02-039/08) and complied with EC regulations (86/609/EEC) for the care and use of laboratory animals. The Extended Methods Form (EMF) for uniform reporting standards [18] can be found in Additional file 1.

### Treatment protocol and groups

Of the 30 rats in which AIA was induced, 10 were treated with morphine (Sigma) at a dose of 2.5 mg/kg in a volume of 250 μL intraperitoneally every testing day 30 minutes prior to behavioral testing. This dose lies in the range of morphine applications usually used for locomotor assessment in arthritic animals [19]. Another group of 10 animals were treated with dexamethasone (Fortecortin Inject; Merck Pharma, Darmstadt, Germany) starting six hours after induction of AIA and received daily injections of 0.3 mg/kg diluted in 300 μL on a five-day injection/two-day no injection schedule to avoid dramatic weight loss (treatment regime adapted from [20]). Following the same treatment scheme as the one we used for dexamethasone, the remaining 10 AIA animals were treated with saline (0.9% NaCl). All animals were assessed for behavioral measures and locomotion twice before induction of arthritis and on days 1, 3, 7, 14 and 21 after induction of AIA.

### Videoradiography

**Figure 1:**
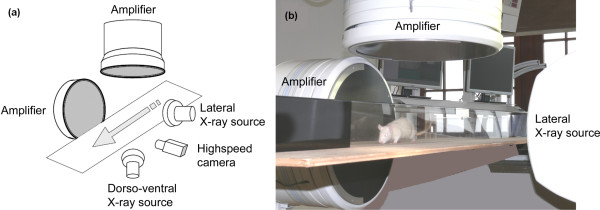
**(A) Sketch of the experimental setup of the X-ray device**. The arrow indicates the direction in which the rat moves while it is filmed by a high-speed camera and X-ray videoradiographs are taken in the lateral and dorsoventral directions. Range of motion was measured using the lateral direction recordings. **(B) **A photograph of the setting is presented for clarity.

On each testing day, animals were left spontaneously walking in a Plexiglas tunnel 2 m in length (height and width 150 mm each). The tunnel was darkened at one end, where the animals went following their instinct to go into the dark. Shortly before reaching the dark end, when the animals walked with constant speed, radiographs were obtained in a lateral perspective using a digital high-speed X-ray system (Neurostar; Siemens, Erlangen, Germany). For the experimental setup used, see Figure 1. Radiographs were recorded with a sampling rate of 500 Hz and a resolution of 1,536 × 1,024 pixels from the amplifier using a high-speed camera (SpeedCam Visario G2; Weinberger Vision GmbH, Erlangen, Germany). For each animal and each testing day, four runs were recorded, corresponding to approximately eight complete step cycles.

**Figure 2:**
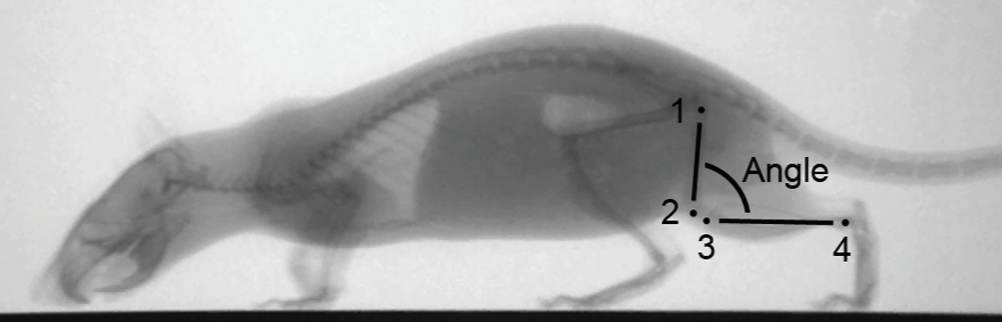
**Landmarks of the rat skeleton used for the determination of the knee joint angle**. Point 1 is the center of the proximal femur epiphysis at the level of the femur neck, point 2 is the center of the distal femur epiphysis at the level of the epicondylus (with points 1 and 2 defining the femoral axis), point 3 is the center of the proximal tibial epiphysis and point 4 is the center of the distal tibial epiphysis at the level of the ankle (with points 3 and 4 defining the tibial axis). The knee joint angle was calculated on the posterior side between the two axes (Angle).

On the X-ray films, the coordinates of defined joint and bone structures were tracked at least in every tenth frame using Simi Motion 3D (Simi Reality Motion Systems GmbH, Unterschleissheim, Germany). The coordinates in the remaining frames were then completed via extrapolation. In particular, the following landmarks were defined: point 1 was the center of the proximal femoral epiphysis at the level of the femoral neck, point 2 was the center of the distal femoral epiphysis at the level of the epicondylus, point 3 was the center of the proximal tibial epiphysis and point 4 was the center of the distal tibial epiphysis at the level of the ankle (Figure 2). Using these landmarks, we calculated the longitudinal axes of the femur and tibia for each frame. The knee joint angle was calculated as the posterior angle between the two bone axes (Figure 2).

**Figure 3:**
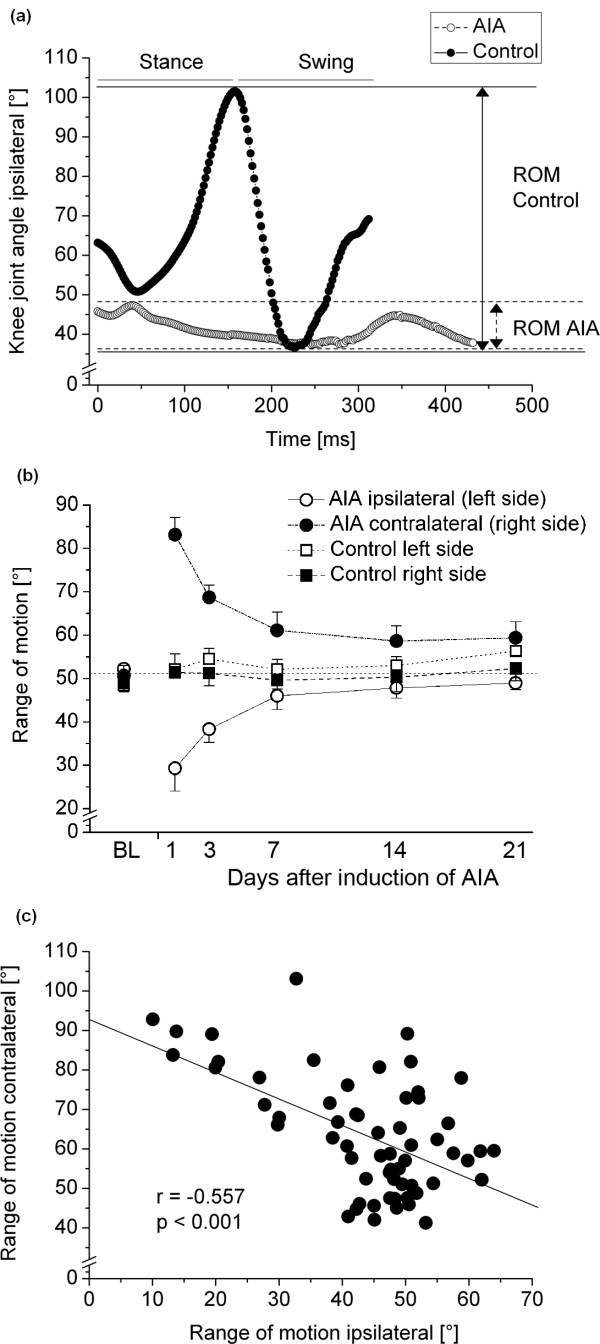
**Locomotion in healthy and arthritic rats**. **(A) **Knee joint angles (*y*-axis) during a complete walking cycle from one representative control animal (filled circles) and from one representative arthritic animal on day 3 after induction of arthritis which did not use the inflamed hindlimb at all (open circles). In the latter animal, the range of motion, defined as the difference between the maximum and minimum knee joint angles during one gait cycle, is reduced. **(B) **Range of motion in the inflamed and the contralateral noninflamed knee joint during the time course of antigen-induced arthritis (AIA) (circles) and in both knee joints of nonarthritic controls during the 21-day observation period (squares). BL : baseline. **(C) **Correlation of range of motion between inflamed and noninflamed knee joints. Each filled circle represents data from a single animal. ROM: range of motion.

The ROM in the knee joint was then obtained from the maximum and minimum joint angles for a complete step cycle as well as separately for stance and swing phases (for further explanation, see Figure 3).

### Pain-related behavior

Primary hyperalgesia at the site of the inflamed knee was assessed using a dynamometer (Correx, Bern, Switzerland) [21]. Increasing pressure was applied to the lateral side of the knee joint at the level of the joint space until the animals attempted to escape or vocalized as described previously [4,22]. The weight force applied to elicit this response was read out in grams. For each animal and each testing day, this test was performed once, since repeated testing might further sensitize the nociceptive apparatus. To prevent tissue damage, a cutoff value of 250 g was defined.

Pain-related guarding behavior of the inflamed hindpaw was assessed by quantification of weight bearing toward the noninflamed hindlimb using an incapacitance tester (Linton Instrumentation, Norfolk, UK). Here animals were placed into a plastic cage with both hindpaws resting on scales. After acclimation to the device when the animal was sitting calmly, the weight force resting on the two scales was obtained and averaged during a 3-second interval, and values from three consecutive measurements were averaged for every testing day. From these values, the relative weight (expressed as percentages) resting on the inflamed hindlimb was calculated (weight on inflamed hindlimb × 100% ? (weight on inflamed hindlimb + weight on noninflamed hindlimb)) as described previously [23,24].

In addition, a guarding score was assessed as described previously [17]: 0 was defined as no guarding; 1 was defined as guarding of the hindlimb after a defined brief, noxious compression of the knee; 2 was defined as visible limping during walking without previous pain stimulus; 3 was defined as no use of the hindlimb with the arthritic knee; and 4 was defined as no movement at all (general morbidity).

### Histology and grading of arthritis

Swelling was assessed by measuring the mediolateral diameter of each knee using a vernier caliper (Mitutoyo, Neuss, Germany). For each animal and each testing day, the relative swelling was calculated by subtracting the diameter of the noninflamed knee from that of the inflamed knee, thus controlling for anatomical knee joint differences between animals.

The histology of the knee joints was assessed on day 21. Rats were deeply anesthetized with 120 mg/kg sodium thiopentone intraperitoneally (Trapanal; Byk Gulden, Konstanz, Germany) and perfused with heparin-enriched PBS and 4.0% phosphate-buffered formalin. The knee joints were removed, skinned, postfixed in formalin, decalcified in 7% AlCl_3 _(in 2.1% HCl and 6% formic acid) for 48 hours, embedded in paraffin, cut into 5-μm-thick frontal sections and stained with hematoxylin and eosin. Two independent observers unaware of the treatment scored the sections (0: no alteration, 1: mild alteration, 2: moderate alteration and 3: severe alteration) for the following alterations: (1) the amount of fibrin exudation and the relative number and density of granulocytes in the synovial membrane and joint space allowed grading of the acute inflammatory reaction; and (2) the relative number and density of infiltrating mononuclear leukocytes in the synovial membrane, the degree of synovial hyperplasia and the extent of infiltration and fibrosis in the periarticular structures allowed grading of chronic inflammation. The scores reflect the sum of all single values for the different criteria. Cartilage and bone destruction were also scored (0: no erosion, 0.5: erosion <10%, focal, 1: 10% to 20%, 1.5: >20% to 40%, 2: >40% to 60%, 2.5: >60% to 80%, and 3: >80% destruction of cartilage and bone in cross-sections) [17,25].

### Statistical analyses

SPSS for Windows version 17.0 software (SPSS, Inc., Chicago, IL, USA) was used for statistical analyses. Data were tested for normal distribution by applying Kolmogorov-Smirnov tests. All parameters except histopathological scores showed normal distribution. For comparison between groups, repeated-measures ANOVA was performed with the between-subjects factor Group (controls versus saline-, morphine- and dexamethasone-treated AIA) and the within-subjects factor Time (baseline and days 1, 3, 7, 14 and 21 after induction of AIA). *Post hoc t*-tests were used to describe differences between groups at different time points whenever ANOVA revealed a significant Group × Time interaction.

Histopathological scores for chronic inflammation and joint destruction were compared between saline, morphine and dexamethasone treatments by using nonparametric Mann-Whitney *U *tests and applying the Bonferroni-Holm correction to account for multiple comparisons.

Kinematic parameters assessed on the basis of videoradiography were correlated with parameters indicating pain-related behavior (primary mechanical hyperalgesia and secondary thermal hyperalgesia) and with factors indicating mechanical disturbance of gait (histopathological scores for inflammation and bone and/or cartilage destruction, knee swelling) using nonparametric Spearman correlation analyses. Significance was accepted at *P *< 0.05.

## Results

All 30 animals in which intraarticular mBSA injections were performed developed signs of AIA, including swelling, pain-related behavior and histopathological changes assessed on day 21, and therefore they were included in the analysis.

### Locomotion in arthritic rats

In nonarthritic control animals, videoradiographic analysis revealed a knee joint angle of approximately 60° at the end of the swing phase (see Figure 3A). After brief flexion, the angle increased to approximately 100° (knee joint extension) when the hindlimb was lifted off the ground (end of stance phase, beginning of swing phase). Following this, the knee joint was flexed to 40° before increasing to 60° for touchdown (see Figure 3A). The mean ROM in this joint, defined as the difference between the maximum and minimum angles, was approximately 60° (see example in Figure 3A). In contrast, arthritic rats that did not use their inflamed hindlimbs (see example in Figure 3A) did not show major movement in the knee joint, but held it at a rather constant angle of around 50° during the complete walking cycle, which cannot be divided into stance and swing phases in these animals, since there is no contact with the floor at any time.

In nonarthritic animals, the ROM remained around baseline values during the complete observation period of 21 days (Figure 3B). However, in arthritic animals, the ROM in the arthritic joint was dramatically reduced in the acute phase of arthritis and slowly recovered to the levels of nonarthritic controls between days 7 and 14 after induction of arthritis (Figure 3B). To maintain locomotion, this diminished movement is compensated by an increased ROM in the contralateral knee joint which mirrors the curve of the ipsilateral joint over time (Figure 3B). Hence, this increased movement appears to be compensational counterregulation of locomotion due to arthritis-induced gait disturbance. This view is further supported by correlational data between the ipsilateral and contralateral knee joints when correlated across all animals (Figure 3C), which shows the data to be significant.

### Effects of anti-inflammatory and antinociceptive treatment on locomotion

**Figure 4:**
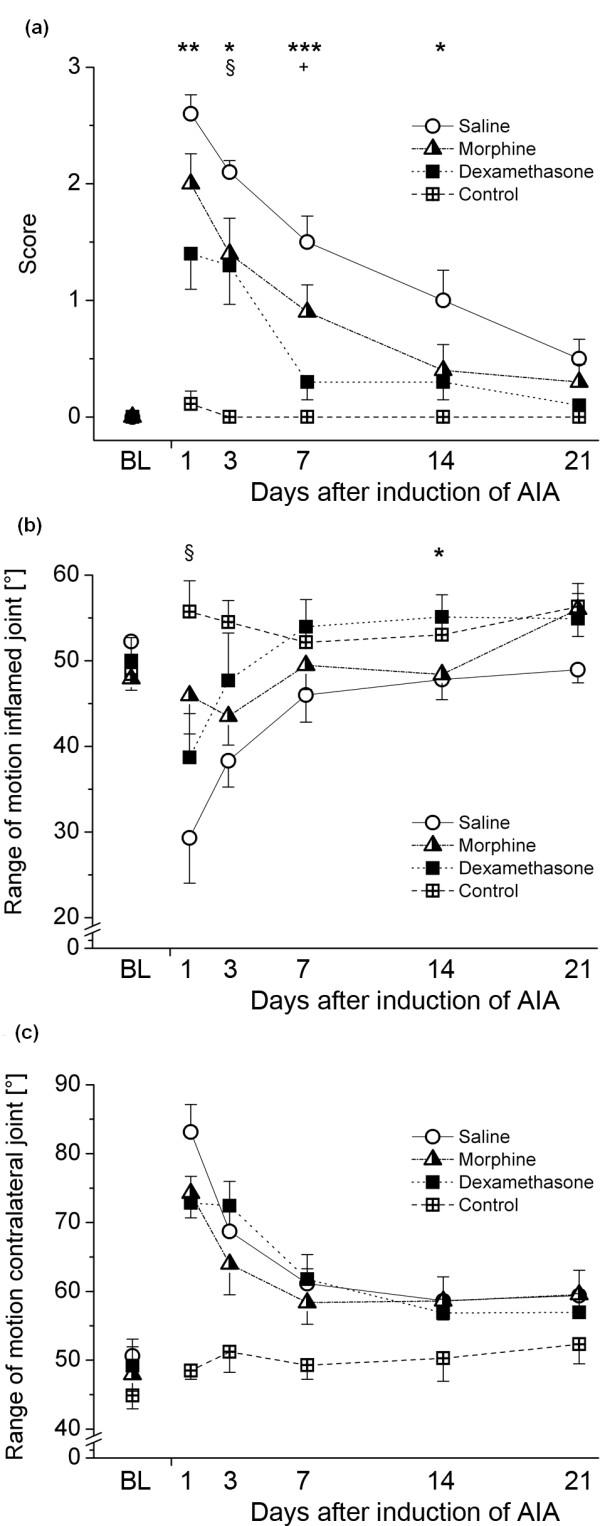
**Locomotor parameters in the different treatment groups over time**. **(A) **Limping score. **(B) **Range of motion (ROM) in the inflamed ipsilateral knee joint. **(C) **ROM in the noninflamed contralateral knee joint. Data are presented as means ± SEM. *Differences between saline and dexamethasone. §Differences between saline and morphine. +Differences between morphine and dexamethasone. Differences between immunized controls and the treatment groups are not presented. *P < 0.05; **P < 0.01; ***P < 0.001.  AIA: antigen-induced arthritis.

A significant Group × Time interaction could be observed for the guarding score [F(15,86) = 3.798; *P *< 0.001]. In particular, AIA animals treated with saline showed visible limping up to the end of the observation period on day 21, while morphine attenuated this sign at all observed time points. Dexamethasone treatment clearly reduced the inflammation-associated locomotor dysfunction in the acute phase (days 1 and 3 after induction) and completely normalized it from day 7 (Figure 4A).

Of the parameters obtained from videoradiography, the ROM of both the inflamed joint [F(15,86) = 1.895; *P *= 0.035] and the contralateral, noninflamed knee joint [F(15,83) = 2.182; *P *= 0.013] showed a significant Group × Time interaction (Figures 4B and 4C). This was also true when considering the stance phase alone (F = 1.889 and *P *= 0.041, F = 2.120 and *P *= 0.016, respectively), but only for the contralateral knee joint in the swing phase (F = 1.528 and *P *= 0.122, F = 1.994 and *P *= 0.025, respectively).

**Figure 5:**
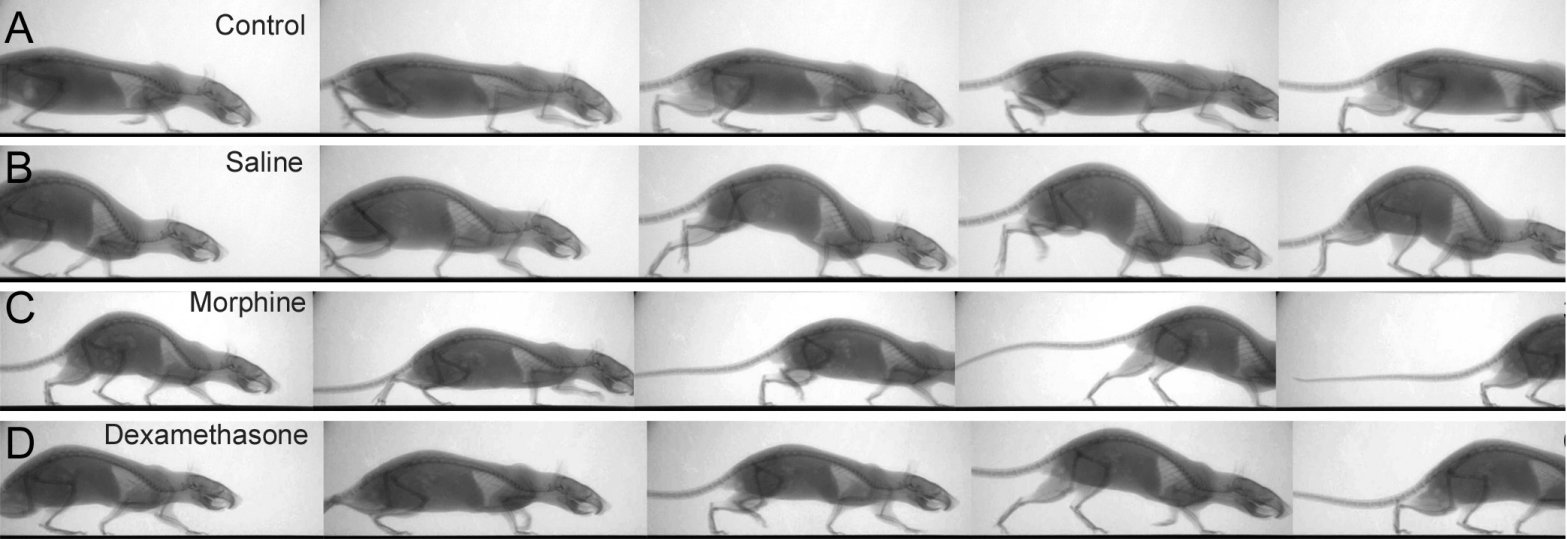
**Walking sequences of representative animals from each treatment group on day 1 after induction of AIA depicting different phases of the gait cycle**. **(A) **Immunized control, no AIA. **(B) **AIA, saline treatment **(C) **AIA, morphine-treatment. **(D) **AIA, dexamethasone treatment.  AIA: anitigen-induced arthritis.

While noninflamed control animals showed a symmetric gait (Figure 5A and Additional file 2), rats treated with saline showed a pronounced change in their overall gait, mainly due to a diminished use of the inflamed hindlimb which was either not used at all for locomotion or was used for very little support only. Representative sequences are shown in Figure 5B (also see Additional file 2). Morphine, and to an even higher degree dexamethasone treatment, partially normalized these alterations (Figures 5C and 5D and Additional file 2).

**Figure 6:**
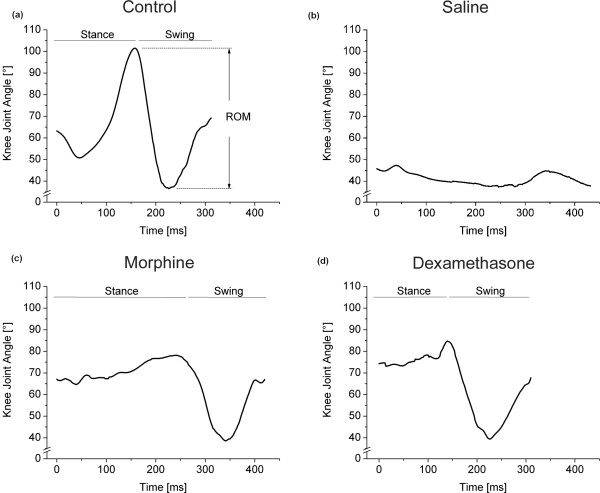
**Change of knee joint angle in the inflamed ipsilateral knee joint during a complete walking cycle measured in representative animals from each treatment group**. **(A) **Immunized control, no AIA. ROM is defined as the difference (in degrees) between the maximum and minimum knee joint angles reached during a complete step cycle. **(B) **AIA, saline treatment. **(C) **AIA, morphine treatment. **(D) **AIA, dexamethasone treatment.  AIA: antigen-induced arthritis; ROM: range of motion.

In the very acute phase, the ROM in the inflamed joint of the saline-treated animals significantly decreased (Figure 6B) in comparison to controls (Figure 6A), while again morphine (Figure 6C) and dexamethasone (Figure 6D) attenuated the inflammation-related reduction in the working range of the joint.

When monitoring the ROM of the inflamed and the noninflamed knee joints over time, we observed that saline-treated animals, as compared to controls, showed the most pronounced decrease in the acute phase, slowly recovering up to day 21 (Figures 4B and 4C). Furthermore, a strong compensational increase of movement was obvious on the contralateral side (Figures 4B and 4C; also see Figure 5 and Additional file 2). Interestingly, the decrease on the ipsilateral side was attenuated by morphine in the acute phase only and to a greater degree by dexamethasone in the chronic phase (Figure 4B), while the compensational movement on the contralateral side appeared to be relatively independent of the underlying treatment, except for the smaller values in the very acute phase on day 1 (Figure 4C).

### Effects of morphine and dexamethasone treatment on pain-related behavior

Significant Group × Time interactions could be observed for primary mechanical hyperalgesia [F(15,86) = 4.304; *P *< 0.001] and weight bearing [F(15,86) = 2.863; *P *< 0.001], but not for secondary hyperalgesia assessed on the contralateral knee [F(15,86) = 1.615; *P *= 0.086].

**Figure 7:**
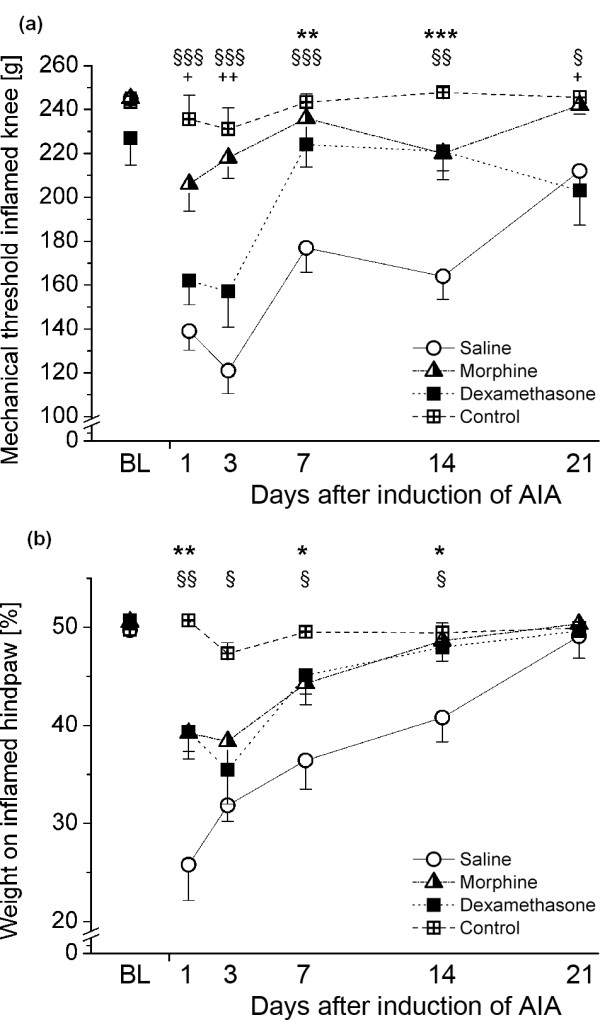
**Pain-related behavior in the different treatment groups over time**. **(A) **Primary mechanical hyperalgesia obtained from mechanical thresholds at the inflamed knee joint. **(B) **Weight-bearing of arthritic animals as indicated by the relative body weight resting on the inflamed hindlimb. Data are presented as means ± SEM. *Differences between saline and dexamethasone. §Differences between saline and morphine. +Differences between morphine and dexamethasone. *P < 0.05; **P < 0.01; ***P < 0.001.  AIA: antigen-induced arthritis.

In particular, weight forces to elicit withdrawal at the site of the inflamed knee joint were dramatically reduced in saline-treated animals and could nearly be prevented by morphine treatment. Dexamethasone did not have a strong antinociceptive effect in the acute phase of AIA, but tended to normalize mechanical thresholds from day 7 after induction of arthritis (Figure 7A).

A shift of body weight toward the noninflamed hindpaw was again most prominent in saline-treated animals, only gradually normalizing until day 21. Here morphine and dexamethasone showed a similar effect, with an attenuated shift in the acute phase and a faster normalization toward baseline values (Figure 7B).

### Effects of morphine and dexamethasone treatment on inflammation and joint destruction

**Figure 8:**
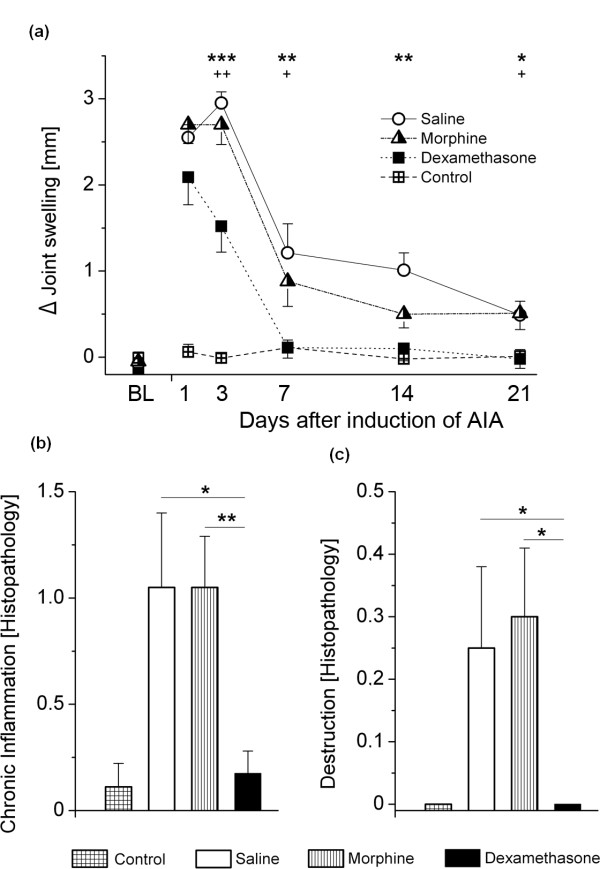
**Severity of inflammation in the different treatment groups**. **(A) **Joint swelling displayed as delta (Δ) between ipsilateral (inflamed) and contralateral (noninflamed) knee joints during the observation period of 21 days. *Differences between saline and dexamethasone. +Differences between morphine and dexamethasone. **(B) **Histopathological scores of joint inflammation. **(C) **Histopathological scores of cartilage and bone destruction. Data are presented as means ± SEM. *P < 0.05; **P < 0.01; ***P < 0.001.  AIA: antigen-induced arthritis.

A significant Group × Time interaction was observed for joint swelling [F(15,86) = 6.734; *P *< 0.001]. Here dexamethasone potently reduced joint swelling, completely abolishing it from day 7 after induction of AIA, while morphine treatment was not different from saline application (Figure 8A).

End point analysis using histopathology revealed no signs of acute inflammation in any of the animals (a single score of 1 was reported in one animal in the saline-treated group) and no difference between treatment groups. Regarding histopathological signs of chronic inflammation, a significant anti-inflammatory effect was apparent for dexamethasone treatment (*P *= 0.009 versus morphine and *P *= 0.019 versus saline), while morphine was again comparable to saline with respect to inflammation (Figure 8B). Joint destruction was likewise reduced in the dexamethasone-treated animals (*P *= 0.031 versus morphine and *P *= 0.047 versus saline) (Figure 8C).

### Correlation analyses

The most stable correlation during the observation period was obtained between ROM on the contralateral side and the guarding score (day 1: *r *= 0.736, *P *= 0.015; day 3: *r *= 0.715, *P *= 0.020; day 7: *r *= 0.699, *P *= 0.024; day 14: *r *= 0.641, *P *= 0.041), indicating that this rather subjective score mainly represents a compensational gain of movement in the contralateral knee. ROM on the inflamed side significantly correlated with weight bearing as assessed using an incapacitance tester in the early phase (day 1: *r *= 0.642, *P *= 0.045; day 7: *r *= 0.656, *P *= 0.039). In the later stages, ROM in the inflamed knee correlated with joint swelling (day 7: *r *= -0.831, *P *= 0.006; day 14: *r *= -0.739, *P *= 0.015; day 21: *r *= -0.667; *P *= 0.035). No further correlations were obtained, and in particular, ROM parameters did not correlate with pain threshold parameters at any stage of arthritis.

## Discussion

In this study, we employed videoradiographic analysis for the first time to describe the ROM in inflamed and noninflamed knee joints of freely moving rats and to relate these data to the inflammatory process and to inflammation-associated pain. The following are the main results that we found. First, in the inflamed knee joint, ROM was dramatically reduced at the acute stage of AIA and slowly recovered to baseline values during the observation period of 21 days. Second, ROM in the inflamed knee was improved by both the anti-inflammatory treatment with dexamethasone (throughout the course of AIA) and the antinociceptive treatment with morphine (at the early stage of AIA), indicating that changes in ROM reflect both inflammation and pain in a stage-dependent manner. Third, correlational analyses showed an association between ROM in the inflamed joint and weight bearing (a parameter of spontaneous or load-dependent pain) in the acute phase, as well as an association between ROM and joint swelling (indicating arthritis severity) in the more chronic stages. Overall, we thus propose that the reduction of ROM in the inflamed joint is an integrative measure of the global severity of arthritis in experimental arthritis models rather than a solely pain-related, inflammation- or joint destruction-related parameter. Fourth, in the contralateral healthy knee joint, ROM was significantly increased, thus compensating for the loss of movement in the inflamed joint.

The examination of knee joint ROM adds a new dimension to the previously used methods of gait analysis in pain and arthritis research. While to date studies have mainly employed temporal or spatial parameters of gait, such as walking speed [5,6,26], duration of stance and swing phases [27,28] and distances or angles between pawprints or footprint pressure [4,19], we have introduced a functional measure of movement which cannot be detected by the methods described previously. This is of particular importance with regard to severe arthritis-related gait changes. As described previously, some animals do not utilize their inflamed hindlimbs, thereby making an assessment based on pawprints, such as pawprint stains or the CatWalk method, impossible [4,8]. In previous studies, data from these most severely affected animals were partly removed from the study, since no objective gait measures could be obtained in early stages of experimental arthritis [4,17].

The relation of ROM parameters to spatial and temporal measures might add to the understanding of disease-related guarding in the future, such as which change in joint movement leads to which gross gait abnormality. It is further worth mentioning that the ROM (in the knee joint) appears to be highly conserved between species [29,30]. In our study, healthy rats displayed ROM values of approximately 60°, a finding which has also been described in studies involving healthy humans [9,13,31]. Knee joint pathology has been shown to reduce this ROM to 15° to 36° in rheumatoid arthritis and to 33° to 40° in osteoarthritis [31], depending on disease severity. Therefore, the results presented here might be transferable to human disease states.

On the basis of the experiments presented here, we cannot determine the exact underlying causes of the decrease in joint movement, since this is related to both joint swelling and measures of pain-related behavior. In previous studies in humans with rheumatoid arthritis or knee joint osteoarthritis, decreased ROM in arthritic joints has also been described [9,10,12,13,32]. In these studies, particular associations have been found between reduced ROM and muscle strength [33] and joint load [13], as well as with Health Assessment Questionnaire scores [9], which reflect disease activity, including muscle strength [34] and pain to a significant degree [35]. Furthermore, associations of knee joint function with joint stiffness and radiographically assessed joint destruction have been suggested [31,36]. In our experiments, dexamethasone-treated animals showed significantly reduced joint swelling and hardly any histopathological signs of chronic inflammation or cartilage and bone destruction, whereas pain-related behavior was still obvious. On the other hand, morphine was capable of significantly attenuating pain-related behavior, but revealed no influence on inflammatory parameters. Therefore, the ROM in the knee joint is unlikely to represent a parameter for inflammation or pain alone, but rather for global arthritis-related disability.

Regardless of the severity of arthritis in the inflamed joint or pharmacological treatment, we observed dramatic counterregulation in the contralateral hindlimb to maintain progression. This was reflected in a significantly increased ROM in these joints. With regard to the respective movement of the affected animals (also see Additional file 2), this finding is due to an increased strain on the contralateral hindlimb to guarantee locomotion. In the most extreme cases, when animals do not use their inflamed hindlimbs at all, the distance to be crossed is overcome by jumping movements of the noninflamed side. Even when the hindlimb is slightly used, however, the noninflamed limb is used to push the body farther away from the surface than in healthy controls, thereby guarding the inflamed limb and decreasing the stance time of the latter.

A somewhat trivial, yet interesting, aspect of this study is the finding that the guarding score which is used to describe the limping and guarding of arthritic animals correlated with ROM on the contralateral side for nearly the complete observation period, but not with ROM in the inflamed knee. This makes sense, since a gain of function is usually easier to observe than a loss of function. Therefore, when subjectively quantifying limping in animals, observers have apparently detected the compensation in the noninflamed joint rather than the actual impairment in the affected knee. This might also be of putative clinical importance, since such compensational movements which outrange the normal functional working parameters of the joint might explain symptoms in neighboring and/or contralateral joints, which are commonly described in both human and animal models (secondary hyperalgesia).

All findings regarding the contralateral hindlimb and thus the assessment of counterregulation could be obtained mainly because we used an asymmetric unilateral inflammation model, namely, AIA. Since only one knee joint is affected in this model, the contralateral joint serves as an internal control. Furthermore, changes in corresponding joints on the other side of the body can be analyzed regarding putative compensational mechanisms. In symmetric models such as adjuvant-induced arthritis or collagen-induced arthritis, compensation is not possible and changes can only be related to healthy controls.

Overall, the ROM observed in both knee joints provides a good screening tool for the estimation of global disease severity in animal models of experimental arthritis and might thus be valuable as an outcome parameter in preclinical assessment of novel antirheumatic or antinociceptive compounds. Particularly in the early phase of arthritis, up to day 3 of AIA, the antinociceptive component can be examined, while over the complete observation period, an estimation of antiarthritic efficacy can be made. Here we validated the respective readout parameters for the model of AIA using anti-inflammatory and antinociceptive pharmacological interventions. Whether these data are unequivocally transferable to other commonly used experimental arthritis models is subject to more detailed analysis in such animals.

## Conclusions

On the basis of the data presented here, pharmacological interventions selectively targeting pain or inflammation discriminate well between groups, whereas the ROM in the inflamed joint is improved to a similar degree by both treatments. Thus, this measure does not represent a solely pain-related, inflammation-related or joint destruction-related parameter. However, the reduction in ROM of the inflamed knee joint represents a valuable parameter to use in the assessment of the global severity of arthritis in experimental arthritis models and appears to be well transferable to human disease states, thereby making it a promising readout parameter for the preclinical assessment of anti-inflammatory agents.

## Abbreviations

AIA: antigen-induced arthritis; ANOVA: analysis of variation; BL: baseline; EC: European Community; EMF: Extended Methods Form; HAQ: Health Assessment Questionnaire; mBSA: methylated bovine serum albumin; PBS: phosphate-buffered saline.

## Competing interests

The authors declare that they have no competing interests.

## Authors' contributions

MKB designed the study, performed the experiments, calculated the statistics, designed the figures, organized the funding and wrote the manuscript. JL performed the experiments. HGS designed and supervised the study. MS designed the study, organized the funding and wrote the manuscript.

## Supplementary Material

Additional file 1 **The Extended Methods Form (EMF) for uniform reporting standards**. Click here for file

Additional file 2 **Radiographic and high-resolution movies showing representative animals from the different treatment groups. **Depicted are an immunized control animal (no arthritis) displaying normal locomotion; an animal with antigen-induced arthritis (AIA) treated with saline displaying very obvious limping, not using the affected hindlimb at all; an animal with AIA and morphine treatment, which guards the affected hindlimb, yet uses it for moving; and an animal with AIA and dexamethasone treatment displaying a pattern similar to that of the morphine-treated animal. Click here for file
