# Supplementary material for: Videoradiographic analysis of the range of motion in unilateral experimental knee joint arthritis in rats
Source: Arthritis Res Ther. 2011 May 27;13(3):R79. doi: 10.1186/ar3342 (PMC3218889; doi:10.1186/ar3342)
Supplement: Additional file 1 — The Extended Methods Form (EMF) for uniform reporting standards. [file ar3342-S1.PDF]

Extended **M**ethods **F**orm

Rice ASC, Cimino-Brown D, Eisenach JC, Kontinen VK, LaCroix-Fralish ML, Machin I, Mogil JS, Stöhr T. Animal models and the prediction of efficacy in clinical trials of analgesic drugs: a critical appraisal and call for uniform reporting standards. *Pain 2008;139(2):241-5*

1. Experimental design

Experiments blinded	Yes
- what	Drug Condition
- how	Observers unaware of treatment (Saline versus morphine versus
Randomization	Yes
Power calculation	Yes
How many data points removed?	1-5
- why	None removed

2. Animals

Age	Young Adult
Sex	Female
Genetic background of mutation	Rat strain: Lewis
Supplier	Charles River, Germany
Weight Range	Rats: 160 - 200 g
Behavioral Abnormalities	None

Any additional comments	

3. Environment

a. *Housing*

Diet	Rodent chow, water a.l.
Bedding	Standard Rodent Bedding
Cage rack ventilation	Not ventilated (std. cage)
Home cage enrichment	None
Habituation time before experiments (in days)	>7 days
Handling frequency before testing	More than once
Cage cleaning frequency	Once per week
# housed per cage	5
House experimental/ control groups together or separately	Same cage
Different species housed in same room	No
Lights on time	7:00
Lights off time	21:00
Procedural anesthetic	None
Post-operative analgesia	No
-Analgesic and dose	

b. *Testing*

# of testers	2
Experience of tester(s)	4-8 years
Lighting intensity (lux)	30
Time testing ends	17:00
Test environment noise	Quiet
Recent calibration of testing equipment	Yes
Cleaning of test equip. between subjects	No
-with	Ethanol
Habituation time before testing (in minutes)	10-30 minutes
Oestrus stage of females	Unknown
Number of animals present in testing room	10
Distance separated	Adjacent in testing device
In visual contact	Yes
Arousal state	Not grooming

Comments on this form to: Andrew Rice *a.rice@imperial.ac.uk*
